# Picoplankton accumulate and recycle polyphosphate to support high primary productivity in coastal Lake Ontario

**DOI:** 10.1038/s41598-019-56042-5

**Published:** 2019-12-20

**Authors:** Jiying Li, Diane Plouchart, Arthur Zastepa, Maria Dittrich

**Affiliations:** 1 Department of Physical and Environmental Sciences, University of Toronto Scarborough, Toronto, ON M1C 1A4 Canada; 2 Canada Center for Inland Waters, Environment and Climate Change Canada, Burlington, ON L7S 1A1 Canada

**Keywords:** Element cycles, Biogeochemistry

## Abstract

Phytoplankton can accumulate polyphosphate (polyP) to alleviate limitation of essential nutrient phosphorus (P). Yet polyP metabolisms in aquatic systems and their roles in P biogeochemical cycle remain elusive. Previously reported polyP enrichment in low-phosphorus oligotrophic marine waters contradicts the common view of polyP as a luxury P-storage molecule. Here, we show that in a P-rich eutrophic bay of Lake Ontario, planktonic polyP is controlled by multiple mechanisms and responds strongly to seasonal variations. Plankton accumulate polyP as P storage under high-P conditions via luxury uptake and use it under acute P stress. Low phosphorus also triggers enrichment of polyP that can be preferentially recycled to attenuate P lost. We discover that picoplankton, despite their low production rates, are responsible for the dynamic polyP metabolisms. Picoplankton store and liberate polyP to support the high primary productivity of blooming algae. PolyP mechanisms enable efficient P recycling on ecosystem and even larger scales.

## Introduction

Phosphorus (P) is an essential element of life. It limits the primary productivity of most freshwater environments and is frequently scarce in many marine systems^1,2^. Phytoplankton can cope with P limitation using various mechanisms^3–5^, which include increasing rates of P uptake and turnover^3,6,7^, releasing enzymes such as alkaline phosphatase to cleave phosphate from extracellular organic compounds^4,8–10^, and substituting P-containing cellular content with other molecules^5,11^. Phytoplankton are also known to accumulate polyphosphate (polyP), a polymer containing three to hundreds of orthophosphate units. The physiological functions of polyP associated with nutrient stringency have been suggested in culture studies^12,13^. PolyP exhibits complex dynamics in cells^13,14^: when P is abundant, cells take up P in excess of what is required for growth to accumulate polyP, a process called “luxury uptake”^13,14^. Cells then can use this polyP to support metabolic processes and growth when P is scarce^13,15^. PolyP also helps cell restoration via a process called “overplus response”: when P-stressed cells experience a P resupply, they take up P rapidly to produce polyP to support recovery from P deficiency^13,15,16^.

PolyP metabolisms in natural environments are scarcely studied, and their potential important roles on ecosystem scales were revealed only recently, including energy reservation, phosphorus recycling and transport^17–24^. PolyP metabolisms in phytoplankton are particularly interesting, as polyP potentially serves as a nutrient reserve for primary productivity and controls the carbon flow. Intriguingly, field observations appear to contradict the view of polyP accumulation as a luxury P storage and/or high-P overplus uptake: the relative accumulation of polyP (the ratio of particulate polyP to total particulate P, polyP:TPP) is lower in relatively high-P systems, whereas phytoplankton accumulate more polyP in low-P systems, termed P “deficiency response”^18,19,21^. In oligotrophic/and or low-P marine environments, phytoplankton also preferentially recycle polyP relative to other forms of particulate P to support P demand^18,19,21^. However, most of the pioneer studies focused on marine systems, many of which are oligotrophic or with low P levels where luxury uptake is unlikely to occur^18,19^, although luxury uptake was inferred in coastal high-P marine waters^20^. Eutrophic inland freshwaters where P is the limiting nutrient have never been investigated. Comparisons between systems may also be challenging because of the potential differences among environments, such as the different threshold of P levels to which polyP responds or the different levels of polyP accumulation under similar conditions^25,26^. The lack of studies in representative freshwater and the scarce of systematic studies describing polyP dynamics under natural conditions hinder our understanding of the diverse polyP functions^13,14^, as well as their roles in phosphorus and carbon biogeochemical cycles on ecosystem and even global scales.

Here, we show that multiple polyP mechanisms co-exist in a natural system with large temporal variability in nutrient levels, representative of many freshwater lakes and coastal oceans. In a high-P eutrophic bay of Lake Ontario, seasonal variability in P levels triggers a dynamic response of polyP. We identify the key polyP accumulators to be different from the phytoplankton that dominate the primary production. Yet polyP metabolisms enable efficient phosphorus recycling, and possibly exchange of phosphorus between communities, allowing primary producers to thrive with continuous access to the limiting nutrient P provided by polyP accumulators.

## Results

### Preferential recycling of polyP in a P limited eutrophic system

**Figure 1:**
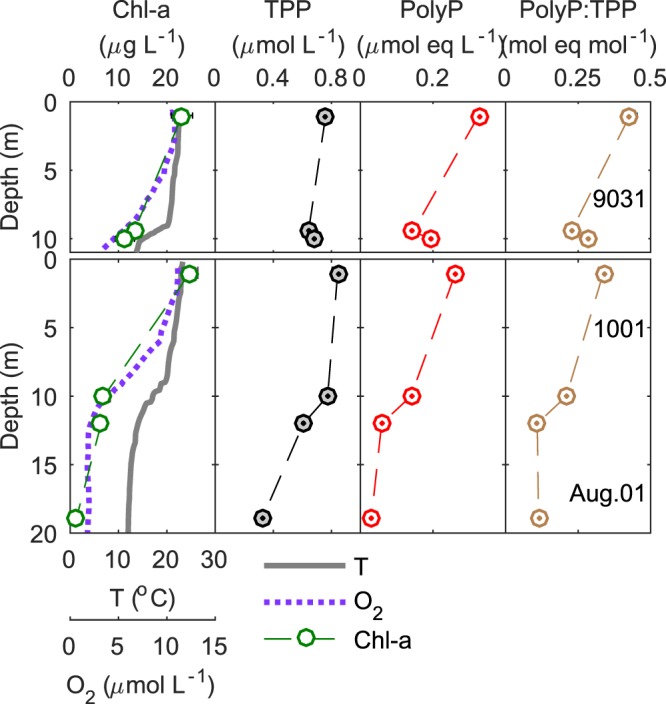
Vertical distributions of temperature (T), dissolved oxygen (O_2_), chlorophyll a (Chl-a), total particulate phosphorus (TPP), polyP, and the ratio of polyP:TPP in the water column of Hamilton Harbour at sites 9031 (top) and 1001 (bottom) (August 1^st^, 2017; see profiles of the entire sampling period in Figs. S2–S4). Measurements were taken at 1 m below surface, one or two locations within the thermocline, and 1 m above the bottom. Standard deviations of the means for three replicate samples are smaller than the marker size.

We investigated the water column of a coastal embayment of Lake Ontario, Hamilton Harbour (Fig. S1), which has been suffering from frequent harmful algal blooms and summer anoxia^27,28^. Total particulate phosphorus (TPP; measured as P in particles >0.2 *µ*m) and polyP concentrations (measured as polyP in particles >0.2 *µ*m) in the surface water of the eutrophic Hamilton Harbour ranged 0.2–1 *µ*mol L^−1^ and 0.05–0.33 *µ*mol eq L^−1^, respectively, considerably higher than those of marine waters (Table 1). The ratios of polyP:TPP in the surface water in Hamilton Harbour varied seasonally between ~ 0.05 and 0.6 mol eq mol^−1^, generally in similar ranges of those in marine environments (Table 1)^18–21^. Figure 1 represents the typical physico-chemical profiles in the summer, showing the vertical distributions of temperature, oxygen, chlorophyll a (Chl-a), TPP, polyP, and polyP:TPP (see Figs. S2–S4 for all profiles for the entire sampling period). PolyP and TPP decreased with depth in the water column, indicating remineralization of organic P and polyP during particle settling^29^. The loss of polyP through particle settling and degrading, calculated as the difference between the surface and the bottom divided by the surface concentrations, averaged 53 ± 13% and 80 ± 13% at sites 9031 and 1001, respectively, higher than the loss of TPP estimated in range of 23 ± 13% and 50 ± 22%, respectively (Table S2; recycling efficiencies of TPP and polyP are significantly different (t-test, *p* < 0.001for both sites)). This results in decreasing ratios of polyP:TPP with depth (Fig. 1), suggesting that polyP was preferentially lost and recycled into the water column as dissolved phase. The attenuation in polyP:TPP was less at the shallower site 9031 because of the weak stratification (Figs. S2–S4). In late fall and winter, the vertical trends in polyP:TPP also disappeared due to mixing (Figs. S2–S4). Whether preferential degradation of polyP also occurred during this period is not known.

**Table 1:** Surface concentrations of TPP, polyP, polyP:TPP, SRP, Chlorophyll a, and APase in Hamilton Harbour compared to other environments.

Site	TPP (nM)	polyP (nM eq)	polyP:TPP (mol eq mol^−1^)	SRP (µM)	Chl-a (µg L^−1^)	APase (nM h^−1^)	Preferential polyP lost	Ref.
Hamilton Harbour, Lake Ontario	200–1000	50–330	0.05–0.6	0.01–0.85	3–28	>100^*^	Yes	This study
Coastal Pacific fjord (Effingham Inlet)	123 ± 1.7	8.6 ± 0.1	0.07	0.5			No	^20^
North Pacific Subtropical Gyre	20	6–8	0.27–0.44	0.06–0.18		0.05–0.13	No	^21^
Temperate western North Atlantic Ocean	130 ± 17	49 ± 18	0.39 ± 0.17	0.1–0.4		ND	No	^19^
Subtropical North Atlantic (Sargasso Sea)	15 ± 1.1	30 ± 9.4	2.0 ± 0.68	0.001–0.025		1.2–4.8	Yes	^19^
Tropical Indian Ocean	10–21	1.2–7.2	0.18–0.42	≤0.03	≤0.05 surface; 0.7–1.0 maximum	0.18–17.3	Yes	^18^

Note: ND = Not detectable; Ref. = Reference; *APase in the present study were measured using a different substrate (*p*-nitrophenyl phosphate (*p*-NPP)) from those of other studies (see Methods). The higher APase measured in the Hamilton Harbour may also be a result of higher biomass: biomass (indicated by TPP concentrations) in Hamilton Harbour was an order of magnitude higher than the North Pacific Subtropical Gyre, the Sargasso Sea, and the Tropical Indian Ocean.

**Figure 2:**
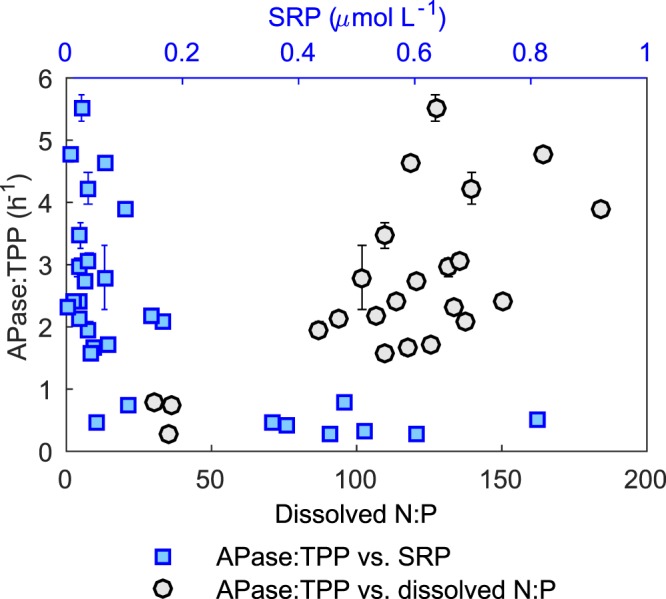
Activity of alkaline phosphatase as functions of soluble reactive phosphorus (SRP) and the ratios of dissolved N:P. Data are from both sites 9031 and 1001 for the entire sampling period, and only include measurements in the surface water. APase is normalized to TPP to account for the variability in biomass. APase:Chl-a and APase plotted against SRP and/or dissolved N:P show similar trends (Fig. S5). Error bars indicate standard deviations of the mean for three replicate samples; error bars are not shown where they are smaller than marker size.

The observation of polyP preferential degradation is consistent with those in some P-depleted parts of oceans (the Sargasso Sea and the tropical Indian Ocean; Table 1)^18,19^. We hypothesize that polyP preferential cycling is regulated by the activities of alkaline phosphatase (APase), a hydrolytic enzyme indicative of phosphorus stress^9,13,30^. In the subtropical North Atlantic Sargasso Sea and the tropical Indian Ocean, dissolved P concentrations were low. This leads to high P stress thus high alkaline phosphatase (APase) activity and preferential polyP degradation^18,19^ (Table 1). On the other hand, in the North Pacific Subtropical Gyre and the temperate North Atlantic where polyP was not preferentially lost, APase activities were much lower (Table 1)^19,21^. In Hamilton Harbour, APase relates negatively with SRP concentrations and positively with the ratios of dissolved N:P in the system (Figs. 2 and S5). Although being eutrophic^28^, Hamilton Harbour is P limited (ratios of dissolved N:P > 100 for dissolved nutrients) and experiences a high level of P stress especially during the period of high productivity in spring and summer. P stress may activate alkaline phosphatase to breakdown and recycle polyP to support high primary productivity. It is not known whether preferential polyP recycling also occurred in other seasons (e.g., during late fall and winter), when the water column was mixed (or weakly stratified). Nevertheless, our results show that preferential recycling of polyP is not limited to oligotrophic systems. It also occurs in P-limiting eutrophic waters. Marine studies demonstrated a lack of preferential polyP recycling in non-P-limited systems^19,21^, and the mechanism in similar freshwater environments needs future investigation.

### PolyP dynamics regulated by multiple mechanisms: deficiency response, luxury uptake, and polyP degradation

**Figure 3:**
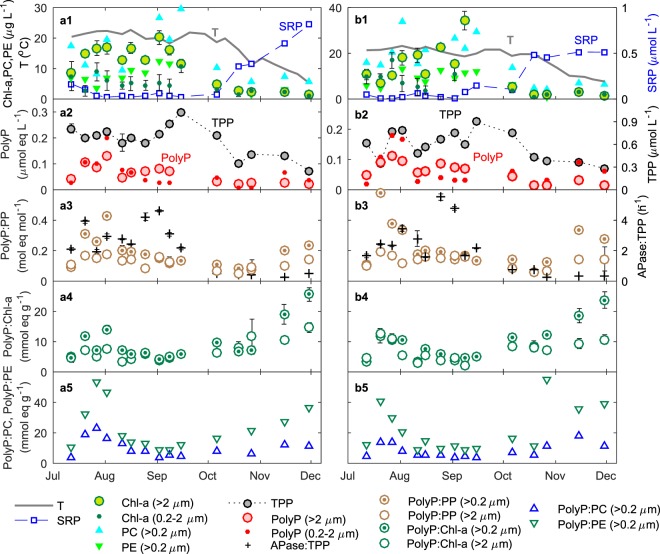
Seasonal variability in the surface water of Hamilton Harbour (1 m). Left and right columns show sites 9031 and 1001, respectively. (**a1,b1**) temperature (T), soluble reactive phosphorus (SRP), chlorophyll a (Chl-a), C-phycocyanin (PC) and phycoerythrin (PE). (**a2,b2**) Total particulate phosphorus (TPP) and polyP. (**a3,b3**) the ratios of polyP:PP ((>0.2 *μ*m polyP: (>0.2 *μ*m PP) and (>2 *μ*m polyP): (>2 *μ*m PP)), and APase*:*TPP (APase and APase:Chl*-*a have similar seasonal variabilities). (**a4,b4**) the ratios of polyP:Chl-a. (**a5**,**a6**) the ratios of polyP:PC and polyP:PE. Error bars indicate standard deviations of the mean for three replicate samples; error bars are not shown where they are smaller than marker size.

The physico-chemical characteristics of the water column of Hamilton Harbour exhibited strong seasonal variability (Fig. 3). The high temperature in the summer and early fall (July – September) resulted in stratification and high primary productivity in the surface water, indicated by peaks of chlorophyll a (Chl-a), and phycobilin pigments C-phycocyanin (PC) and phycoerythrin (PE) that are characteristic of cyanobacteria (Fig. 3a1,b1). Pigment concentrations decreased as the surface temperature dropped during winter. Concentrations of SRP in the surface water were low during the summer and early fall due to high P uptake and increased to as high as >0.5 *µ*mol L^−1^ during winter after the breakdown of stratification and the mixing of the deep nutrient-rich water to the surface (Fig. 3a1,b1). PolyP was high in summer (Fig. 3a2,b2), generally corresponding to peaks of pigments and TPP (Fig. 3a2,b2). The ratios of polyP:TPP (measured in the size fraction of >0.2 *μ*m) exhibited two distinct periods of enrichment, between mid-July and August (summer) and after mid-November (winter) (Fig. 3a3,b3). Similar patterns were also observed in the ratios of polyP to phytoplankton pigments (polyP:Chl-a, polyP:PC, and polyP:PE; Fig. 3a4,b4, 3a5,b5).

The seasonal variability of polyP in the water column of the Hamilton Harbour represents planktonic physiological responses to the dynamics of nutrients. Enrichment of polyP in summer was likely a result of P deficiency response during this period of high productivity. Phosphorus deficiency activates the regulatory genes (Pho regulon) and trigger polyP enrichment by preferentially accumulating polyP over other cellular P forms^9,31,32^, or preferentially degrading other non-polyP P pools (e.g., breaking down of DNA and RNA, and substitution of phospholipids)^5,19,33^. PolyP accumulation as P deficiency response plays important roles in the biogeochemical cycling of phosphorus in oligotrophic marine environments^17–19,21,34^. P deficiency response was also observed in periphyton communities in an oligotrophic freshwater stream^35^. Our results suggest that this mechanism is not limited to oligotrophic systems. In eutrophic Hamilton Harbour, despite P levels being much higher on average compared to oligotrophic systems (Table 1), P stress may occur during periods of high primary productivity leading to polyP accumulation in plankton as P deficiency response. P stress might have triggered the high activity of APase (Fig. 3a3,b3) and resulted in preferential degradation of polyP (Figs. 1, S2–S4, and Table S2), which actively retains bioavailable P in the system to sustain the growth of primary producers in this eutrophic system.

Increase of polyP quotas (polyP:TPP, polyP:Chl-a, polyP:PC and polyP:PE) in the winter, however, was caused by a different mechanism. Increase of SRP in the surface water during winter mixing might have triggered an “overplus” response: when P-stressed cells experience a sudden increase of P supply, they take up P rapidly and produce a high level of polyP to support a restoration of phosphorus supply^13,15,16^. However, the high polyP:TPP ratios resulted from overplus uptake may not be maintained once the organisms are adjusted to the high ambient P levels. Our prior culture experiment suggested that cyanobacteria recovered from overplus response in less than 5–10 days, even though ambient P level was still high (>20 *μ*mol L^−1^)^13^. The persisting high polyP:TPP may be rather explained by another mechanism, luxury uptake: under conditions of P supply in excess, plankton take up P exceeding growth demand to accumulate polyP as P storage^13,36^. SRP concentrations remained high during the whole period of winter sampling (mid-October to December), thus likely sustain P luxury uptake. Although we do not know how fast plankton recovers from overplus uptake in natural environments, the high polyP in winter was likely a result of luxury uptake, possibly in combination with overplus response.

**Figure 4:**
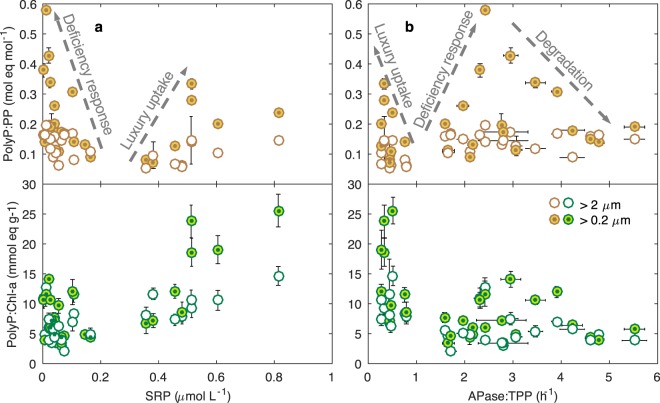
PolyP ratios (polyP:PP and polyP:Chl-a) in different size fractions plotted against soluble reactive phosphorus (SRP) and activity of alkaline phosphatase (APase:TPP, h^−1^; Fig. S6 plots polyP ratios against APase (*μ*mol L^−1^ h^−1^) and APase:Chl-a (*μ*mol h^−1^ mg^−1^) showing similar trends). Data are from both sites 9031 and 1001 for the entire sampling period, and only include measurements in the surface water. Error bars indicate standard deviations of the mean for three replicate samples; error bars are not shown where they are smaller than marker size.

The variabilities of polyP quotas across the gradients of SRP and APase provide more insights into the polyP dynamics regulated by various mechanisms (Fig. 4; see Fig. S6 for more data). High ratios of polyP at low SRP concentrations suggested P deficiency responses, while the increase of polyP ratios under high SRP concentrations (>0.4 *µ*mol L^−1^) were indicative of luxury and/or overplus uptake (Fig. 4a). Similarly, the high polyP ratios at low APase levels indicated polyP luxury accumulation when P was abundant (Fig. 4b); the increase of P stress (APase) led to polyP accumulation via P deficiency responses (Fig. 4b). Further increase of APase (more severe P stress), on the other hand, led to decreases of polyP ratios: both polyP:PP and polyP: Chl-a decreased when APase was larger than ~2.5 h^−1^ (Fig. 4b). This suggests that under acute P stress, polyP became a P reserve and was degraded to provide SRP for plankton survival, consistent with what we found in a culture study of cyanobacteria^13^. Degradation of polyP was not observed in the ultra-oligotrophic Sargasso Sea where P concentrations were lower than the eutrophic Hamilton Harbour^19^ (Table 1). APase in the Sargasso Sea was in the order of 0.08–0.32 h^−1^ (Table 1), lower than >3 h^−1^ in Hamilton Harbour in late summer when polyP decreased (Fig. 3; APase is normalized to TPP for comparison between two systems). Therefore, the Sargasso Sea might not have reached the level of high APase (P stress) that would lead to polyP degradation. The comparison between the two systems should be interpreted with caution, however, because of the different substrates used to measure APase. The concentration of P (and/or the APase level) at which P stress is severe enough for polyP to be degraded is not known and may depend on factors such as nutrient stoichiometry and the plankton communities. The plankton communities in the chronically low-P Sargasso Sea might be more adapted to P stress and can maintain polyP enrichment (high polyP:TPP). Nevertheless, our results show that in natural systems, accumulated polyP can serve as a P reserve when P levels become too low.

The level of polyP:TPP due to luxury uptake, although being higher than normal, were generally lower than the elevated polyP:TPP triggered by P deficiency (Figs. 3a3,b3, and 4). This suggests that the lower polyP:TPP in high-P marine systems compared to low-P systems^19,21^ does not exclude the possibility of luxury uptake under high-P conditions^20^, because the signal of luxury uptake may have been concealed. Our data collected within a wide range of P levels in the seasonally dynamic eutrophic Hamilton Harbour overcame this challenge and revealed diverse and dynamic polyP mechanisms in play in a single system. This advances our understanding of the roles of polyP in plankton physiology in aquatic environments: in addition to P deficiency response^10,19^, plankton also respond to P excess by accumulating polyP via luxury uptake and/or overplus response. The accumulated polyP can serve as a P reserve and be liberated upon acute P stresses even in eutrophic systems.

### Picoplankton contributes predominately to polyP dynamics

Primary productivity during summer algal blooming in Hamilton Harbour was composed primarily by larger size phytoplankton (>2 *µ*m), including eukaryotic algae and some cyanobacteria (e.g., filamentous)^37^, which is a general characteristic of eutrophic systems^37^. The dominant phytoplankton taxonomic groups in the water column of Hamilton Harbour include eukaryotic diatoms, dinoflagellates, chlorophytes, chrysophytes, and cryptophytes as well as prokaryotic cyanobacteria^37^. The dominant genera of cyanobacteria, one of the major phylum of primary producers in the bay, include *Planktonthrix, Synechococcus, Chroococcidiopsis, Cuspidothrix, Pseudanabaena, Microcystis, Limnococcus*, and *Arthrospira* (typically contributing to >90% of cyanobacteria and ~9% of total prokaryotes in sequence counts; Fig. S7). Interestingly, during the periods of enhanced polyP accumulation, polyP in picoplankton (<2 *µ*m) was higher than in larger size phytoplankton (Fig. 3). The seasonal dynamics of polyP in picoplankton was stronger with higher fluctuations in both polyP:PP and polyP:Chl-a compared to those of the larger size fractions (polyP:PP and polyP:Chl-a of particles >2 *μ*m; Figs. 3 and 4). This suggests that picoplankton are more sensitive to ambient P levels and strongly respond by adjusting their polyP quotas. Picoplankton primarily consisted of picocyanobacteria and heterotrophic bacteria (Fig. S7). Both groups are known to accumulate polyP^13,38^. Particularly, they are suggested to take up more P, have higher rates of P uptake, and show more pronounced seasonal variability in P content compared to larger size algae, likely because of their higher affinity to P on account of their higher surface-to-volume ratios^39–42^. Our finding that microbial communities of different size-fractions have different polyP metabolisms and dynamics is consistent with culture studies showing taxonomic variability in polyP metabolisms^13,25^. This might help explain the variations in polyP observed in marine systems. For example, the communities with greater polyP storage capacity might better survive in environments with chronical low-P levels, such as the ultra-low P Sargasso Sea, while in the temperate North Atlantic communities might be less responsive in terms of polyP metabolism^19^. The variability in polyP among systems, therefore, might be due to both differences in biogeochemistry and community compositions. To better understand polyP dynamics in natural aquatic systems and its contributors, we propose future work to quantify cell-specific polyP and investigate polyP dynamics under changing community structures.

Although changes in microbial communities would lead to variations in polyP as discussed above, the polyP dynamics in Hamilton Harbour is largely due to physiological responses to changing P level rather than shifts in taxonomy. We analyzed the taxonomic data using Nonmetric Multidimensional Scaling (NMDS; Fig. S8) and fit polyP:TPP and other environmental parameters (Temperature, SRP, NO_3_^−^ and NO_2_^−^, NH_4_^+^) onto the ordination (Fig. S8). PolyP:TPP does not significantly explain the variation in the taxonomy (*p* > 0.1; Fig. S8). Therefore, the dynamics of polyP accumulation in Hamilton Harbour cannot be explained solely by taxonomic shifts but likely also due to physiological shift, that is, polyP metabolisms responding to variations in P levels.

## Discussion

**Figure 5:**
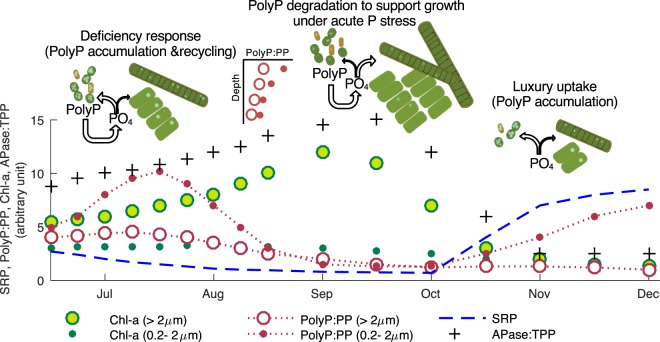
Illustrations of polyP metabolisms and their roles in phosphorus biogeochemical cycling (data points are conceptual). In early-mid summer SRP concentrations decrease due to increasing P uptake, leading to P stress (high APase) and polyP enrichment in picoplankton as a P deficiency response. This enables efficient P recycling due to preferential degradation of polyP, keeping bioavailable P in the water column to support primary productivity. During the late summer peak of primary production, acute P stress (elevated APase) leads to polyP degradation that provides P for blooming algae. High SRP concentration in winter triggers P luxury uptake, an important P storage mechanism that may be beneficial for algal growth in subsequent spring and summer. All values in the schematic plots are in arbitrary units.

Our work reveals multiple and variable polyP mechanisms in aquatic systems that efficiently recycle phosphorus to support diverse microbial communities and meet ecosystem-scale nutrient demand. This is illustrated in Fig. 5. High nutrient uptake in summer leads to P stress, which triggers the enrichment of polyP in picoplankton as a P deficiency response. It also leads to efficient P recycling due to the preferential degradation of polyP. In late summer and early fall during peak primary production, the accumulated polyP in picoplankton is degraded to become available for larger-size blooming algae. The ability of plankton to accumulate polyP as a P reserve may be crucial in regulating primary productivity in P-limiting eutrophic systems. In P-limiting systems, seasonal nutrient dynamics and productivity are sometimes out of phase – algal blooms occur when the supply of P is low. This is true in Hamilton Harbour, where cyanobacteria blooms often occur in late summer despite significant reductions in external inputs of both N and P^43^ and lower SRP concentration due to higher uptake (Figs. 3 and 5). Although other conditions are also important for harmful algal bloom, including temperature, light and other co-limiting nutrients^44–46^, blooming arguably requires available P. A common explanation to this paradox is that rapid P turnover in the surface euphotic zone recycles P to meet the high P demand^6,7^. Our observation of the preferential degradation of polyP supports this theory. Also, plankton can take advantage of high P conditions by accumulating polyP as a P reserve, even though other factors may not be favorable for growth. The P stored in the form of polyP can then be used once conditions become more suitable for growth (e.g., higher temperature). This finding provides an alternative/complementary explanation for the paradox of high productivity under low P conditions.

Picoplankton, including the picocyanobacteria and heterotrophic bacteria, have received less attention in eutrophic systems compared to the intensive focus on blooming phytoplankton^27,43^. Our results show that while the larger-size phytoplankton bloom, picoplankton store P as polyP and metabolize it, likely an important P cycling mechanism for the whole ecosystem: they become nutrient providers and support growth of blooming phytoplankton by rapidly recycling polyP. Moreover, we hypothesize PolyP metabolisms being important in metabolic coupling of cyanobacteria to heterotrophic bacteria. Cyanobacteria-bacteria synergism is common among bloom-forming genera^45^ and exchange of P has been observed^47–50^. Due to their high P affinity compared to larger algae, bacteria are usually energy-limited (organic carbon limited) rather than P-limited and dependent on algal organic exudates for their energy supply^40,51^. At the same time, bacteria excrete P to support algal growth and energy production from which they can benefit^42,48,52^. This synergic mechanism is ecologically beneficial because it enables the plankton’s continuous access to the limiting nutrient P, which can be used for both heterotrophic bacterial production and primary production even when ambient P is low. The metabolic coupling among microbial communities via polyP mechanisms needs further investigation.

Picoplankton accumulating more polyP as luxury uptake during the less productive winter season may be ecologically important. Winter ecology in aquatic systems has shown to affect summer primary productivity, even though the mechanisms and the magnitudes of such interactions remain unclear^53^. Our results suggest that winter conditions (e.g., higher P, colder water, and lower light) favor polyP storage in picoplankton. Whether this is a universal winter phenomenon beneficial for phytoplankton growth in the subsequent productive period (e.g., during spring blooms) deserves further investigations.

In conclusion, in a eutrophic bay of Lake Ontario, plankton accumulate and metabolize phosphorus polymers polyphosphate to cope with phosphorus limitation. Planktonic cells accumulate polyP as storage under high phosphorus conditions to overcome future phosphorus stresses. PolyP enrichment also alleviates phosphorus limitation, because polyP is more readily recycled than other phosphorus compounds thus retain bioavailable phosphorus in the system. Notably, small-size picoplankton, which are minor contributors to primary productivity, are responsible for the strategic polyP metabolisms. By storing and liberating polyP, picoplankton serve as phosphorus bank to support the primary productivity, predominantly that of the bloom-forming algae. These findings advance our knowledge beyond previously observed polyP mechanisms. The diverse polyP mechanisms enable efficient P recycling and support P demands of various planktonic communities. The mechanisms may have strengthened the phosphorus-carbon coupling, and have implications for similar systems such as many freshwater inland lakes and coastal eutrophic systems experiencing strong dynamics in nutrient availability.

## Methods

### Study sites and sampling

Water samples were collected from two sites (sites 9031 and 1001 with maximal water depths of 12 m and 24 m, respectively) weekly/biweekly from mid-summer (July) to early winter (end of November) of 2017 (Table S1, Fig. S1). Vertical profiles of temperature and dissolved oxygen concentrations were measured using YSI 6600 V2 Multi-parameter Sonde. Water samples were collected using a 10 L Niskin sampler at selected depths – 1 m below surface, one or two locations within the thermocline, and 1 m above the bottom. Suspended particles were collected using 0.2 *μ*m GTTP (Millipore), and 2.0 *μ*m TTTP or 1.2 *μ*m RTTP (Millipore) filters (Table S1), within 24 hours of sample collection. For analyses of pigments, sample processing (e.g., filtration) was conducted in dark. The water passed through 0.2 μm filters were stored frozen at −20 °C until further analyses of soluble reactive phosphorus (SRP). For samples collected in anoxic waters, additional filtrates were also acidified with 1% 6 N hydrochloride acid (HCl) to prevent oxidation of reduced iron and formation of iron particles that scavenge SRP. Particulate samples collected on filters were stored frozen at −80 °C before analyses.

### Chemical analyses

We measured sized-fractionated particular phosphorus (PP) and particulate polyP (polyP hereafter; sizes of 2 *µ*m and 0.2 *µ*m; Table S1). Particles collected on 0.2 *µ*m filters were considered including both prokaryotic and eukaryotic plankton of >0.2 *µ*m, and its difference from the particles collected on 2 *µ*m filters was attributed to the small size fraction picoplankton (0.2–2 *µ*m)^54^. Particulate phosphorus was extracted using persulfate digestion followed by analyses of soluble reactive phosphorus (SRP) in extracts spectrometrically using molybdenum blue method^55,56^. PP measured on 0.2 *µ*m filters was considered total particulate phosphorus (TPP). SRP was also determined in the filtrates of 0.2 *µ*m filters.

PolyP in particles was extracted using boiling and enzymatic digestion^57^, then separated from particles by centrifugation (×12000 g, 10 min), and determined for concentrations fluorometrically at an excitation wavelength of 415 nm and an emission wavelength of 550 nm^57,58^ after incubation with 2,6- diamidino-2-phenylindole (DAPI)^57^. Fluorescence readings for polyP were calibrated using standard solutions of synthetic polyP-60 (chain length of 60 phosphates; gifted from Dr. Toshikazu Shiba, Matsumoto University, Japan). Because the methods only give a relative measure of polyP concentration, we use the convention units reported for polyP methods, which is micromole equivalents of P per liter (*µ*mol eq L^−1^) for polyP and mole equivalents of polyP per mol of PP (mol eq mol^−1^) for polyP:PP.

Subsamples of filters were extracted for chlorophyll using 90% aqueous acetone (v/v) as solvent^59^. Samples were disrupted and homogenized using vortex (5 s) then sonication in ice bath (Qsonica Q125 probe sonicator with probe diameter of 0.32 cm, for 20 s on a pulse mode (1 s on 1 s off) at 50 W), followed by 12 hours incubation at 4 °C in dark then centrifuged (×12000 g, 10 min) to remove particles and filter debris prior to measurements of Chl-a concentrations^59^. Chl-a concentrations were determined fluorometrically at an excitation wavelength of 430 nm and an emission wavelength of 663 nm^60^. The water-soluble phycobilin pigments C-phycocyanin (PC) and phycoerythrin (PE), characteristic of cyanobacteria, were extracted in phosphate buffer using a freeze-thaw cycle followed by sonication in an ice bath and extraction at 4 °C in dark for 24 hours^61,62^ and measured spectrometrically^63,64^.

### Activity of alkaline phosphatase

Activity of alkaline phosphatase (APase) was determined in the unfiltered samples immediately upon samples arrival at the laboratory (within 24 hours after sample collection) using a colorimetric assay^9^. Briefly, 1 mL of samples was buffered with Tris-HCl solution (pH 8.5) with the addition of *p*-nitrophenyl phosphate (*p*-NPP) as a substrate, incubated at 25 °C for ~24 hours. The samples were centrifuged (×12000 g, 10 min) to remove the suspended particles and measured the concentrations of the reaction product *p*-nitrophenol (*p*-NP) spectrophotometrically at 410 nm. APase activity was calculated as *μ*mol *p*-NPP hydrolyzed (*p*-NP or PO_4_ produced) per hour per liter of water (*μ*mol P h^−1^ L^−1^). The substrate used in this study, *p*-NPP, was different from those of other studies in marine environments, typically the 6,8-difluoro-4-methylumbelliferyl phosphate or 4-methylumbelliferyl phosphate (MUF-P; see Table 1), which is more sensitive than *p*-NPP. Comparison of results obtained by the different methods should be interpreted with caution. While production of extracellular phosphatase is not a general response to P starvation in phytoplankton, our measurement is not species-specific as the enzyme labeled fluorescence technique^65^.

### DNA extraction, sequencing, and statistical analysis

Particulate samples that collected on 0.2 *μ*m GTTP polycarbonate filters were extracted for DNA using the DNeasy Powerbiofilm kit (Qiagen, Hilden, Germany). Briefly, the V3-V4 regions of 16S rRNA gene were amplified using the HotStarTaq Plus Master Mix Kit (Qiagen, Hilden, Germany) with primers 341 F (CCTACGG GNGGCWGCAG) and 805R (GACTACHVGGGTATCTAATCC)^66^. Post-amplification PCR products were checked for quality in 2% agarose gel, pooled and purified using calibrated Ampure XP beads. Amplicon sequencing was performed at Mr. DNA (http://www.mrdnalab.com, Shallowater, TX, USA) using a MiSeq (Illumina). Sequences data were processed using Mr. DNA analysis pipeline (Mr. DNA, http://www.mrdna.com, Shallowater, TX, USA) to generate operational taxonomic units (OTUs; 97% similarity). Final OTUs were taxonomically classified using BLASTn against a curated database derived from RDPII and NCBI (www.ncbi.nlm.nih.gov, http://rdp.cme.msu.edu). Relative abundances of phyla and genera were generated using the phyloseq package^67^. We used the “vegan” package in *R* to compute Nonmetric Multidimensional Scaling (NMDS) of the bacterial community and fit environmental variables to the ordination^68^.

## Supplementary information

Supporting information

## Data Availability

The data supporting the findings of this study are available within this article and its Supporting Information, and all additional datasets generated during the current study are available from the corresponding author on reasonable request.

## References

[1] Karl DM (2014). Microbially mediated transformations of phosphorus in the sea: new views of an old cycle. Annual Review of Marine Science.

[2] Hecky RE, Kilham P (1988). Nutrient Limitation of Phytoplankton in Fresh-Water and Marine Environments - a Review of Recent-Evidence on the Effects of Enrichment. Limnology and Oceanography.

[3] Riegman R, Stolte W, Noordeloos AA, Slezak D (2000). Nutrient uptake and alkaline phosphatase (EC 3: 1: 3: 1) activity of Emiliania huxleyi (Prymnesiophyceae) during growth under N and P limitation in continuous cultures. Journal of phycology.

[4] Lomas MW, Swain A, Shelton R, Ammerman JW (2004). Taxonomic variability of phosphorus stress in Sargasso Sea phytoplankton. Limnology and Oceanography.

[5] Van Mooy BA (2009). Phytoplankton in the ocean use non-phosphorus lipids in response to phosphorus scarcity. Nature.

[6] Colman AS, Blake RE, Karl DM, Fogel ML, Turekian KK (2005). Marine phosphate oxygen isotopes and organic matter remineralization in the oceans. Proceedings of the National Academy of Sciences of the United States of America.

[7] Li J, Bai Y, Bear K, Joshi S, Jaisi D (2017). Phosphorus availability and turnover in the Chesapeake Bay: Insights from nutrient stoichiometry and phosphate oxygen isotope ratios. J Geophys Res-Biogeo.

[8] Duhamel S, Dyhrman ST, Karl DM (2010). Alkaline phosphatase activity and regulation in the North Pacific Subtropical Gyre. Limnology and Oceanography.

[9] Adams MM, Gomez-Garcia MR, Grossman AR, Bhaya D (2008). Phosphorus Deprivation Responses and Phosphonate Utilization in a Thermophilic Synechococcus sp from Microbial Mats. Journal of Bacteriology.

[10] Dyhrman ST (2012). The Transcriptome and Proteome of the Diatom Thalassiosira pseudonana Reveal a Diverse Phosphorus Stress Response. PloS one.

[11] Geider RJ, La Roche J (2002). Redfield revisited: variability of C: N: P in marine microalgae and its biochemical basis. European Journal of Phycology.

[12] Rao NN, Gomez-Garcia MR, Kornberg A (2009). Inorganic Polyphosphate: Essential for Growth and Survival. Annu Rev Biochem.

[13] Li J, Dittrich M (2019). Dynamic polyphosphate metabolism in cyanobacteria responding to phosphorus availability. Environmental microbiology.

[14] Jensen, T. E. & Sicko-Goad, L. *Aspects of phosphate utilization by blue-green algae*. (US Environmental Protection Agency, Office of Research and Development, Corvallis Environmental Research Laboratory, 1976).

[15] Sicko-Goad LM, Jensen TE (1976). Phosphate-Metabolism in Blue-Green-Algae .2. Changes in Phosphate Distribution during Starvation and Polyphosphate-Overplus Phenomenon in Plectonema-Boryanum. Am J Bot.

[16] Jensen TE, Sicko LM (1974). Phosphate-Metabolism in Blue-Green-Algae .1. Fine-Structure of Polyphosphate Overplus Phenomenon in Plectonema-Boryanum. Can J Microbiol.

[17] Orchard ED, Benitez-Nelson CR, Pellechia PJ, Lomas MW, Dyhrman ST (2010). Polyphosphate in Trichodesmium from the low-phosphorus Sargasso Sea. Limnology and Oceanography.

[18] Martin P (2018). Particulate polyphosphate and alkaline phosphatase activity across a latitudinal transect in the tropical Indian Ocean. Limnology and Oceanography.

[19] Martin P, Dyhrman ST, Lomas MW, Poulton NJ, Van Mooy BAS (2014). Accumulation and enhanced cycling of polyphosphate by Sargasso Sea plankton in response to low phosphorus. Proceedings of the National Academy of Sciences of the United States of America.

[20] Diaz Julia M., Ingall Ellery D., Snow Samuel D., Benitez-Nelson Claudia R., Taillefert Martial, Brandes Jay A. (2012). Potential role of inorganic polyphosphate in the cycling of phosphorus within the hypoxic water column of Effingham Inlet, British Columbia. Global Biogeochemical Cycles.

[21] Diaz JM (2016). Polyphosphate dynamics at Station ALOHA, North Pacific subtropical gyre. Limnology and Oceanography.

[22] Diaz J (2008). Marine polyphosphate: A key player in geologic phosphorus sequestration. Science.

[23] Möller L (2019). Sulfurimonas subgroup GD17 cells accumulate polyphosphate under fluctuating redox conditions in the Baltic Sea: possible implications for their ecology. The ISME journal.

[24] Schulz-Vogt, H. N. *et al*. Effect of large magnetotactic bacteria with polyphosphate inclusions on the phosphate profile of the suboxic zone in the Black Sea. *The ISME journal*, 1 (2019). 10.1038/s41396-018-0315-6 PMC6474215 30643197

[25] Mazard S, Wilson WH, Scanlan DJ (2012). Dissecting the Physiological Response to Phosphorus Stress in Marine Synechococcus Isolates (Cyanophyceae). Journal of phycology.

[26] Mateo P, Douterelo I, Berrendero E, Perona E (2006). Physiological differences between two species of cyanobacteria in relation to phosphorus limitation. Journal of phycology.

[27] Jonlija, M. Assessment of toxic cyanobacterial abundance at Hamilton Harbour from analysis of sediment and water (University of Waterloo, 2014).

[28] Munawar M (2017). Phytoplankton ecology of a culturally eutrophic embayment: Hamilton Harbour, Lake Ontario. Aquatic Ecosystem Health & Management.

[29] Li J (2017). Water column particulate matter: A key contributor to phosphorus regeneration in a coastal eutrophic environment, the Chesapeake Bay. J Geophys Res-Biogeo.

[30] Huang R, Wan B, Hultz M, Diaz JM, Tang Y (2018). Phosphatase-mediated hydrolysis of linear polyphosphates. Environmental science & technology.

[31] Ault-Riche D, Fraley CD, Tzeng CM, Kornberg A (1998). Novel assay reveals multiple pathways regulating stress-induced accumulations of inorganic polyphosphate in Escherichia coli. Journal of Bacteriology.

[32] Rao NN, Liu SJ, Kornberg A (1998). Inorganic polyphosphate in Escherichia coli: the phosphate regulon and the stringent response. Journal of Bacteriology.

[33] Elser J (2003). Growth rate–stoichiometry couplings in diverse biota. Ecology Letters.

[34] Temperton Ben, Gilbert Jack A., Quinn John P., McGrath John W. (2011). Novel Analysis of Oceanic Surface Water Metagenomes Suggests Importance of Polyphosphate Metabolism in Oligotrophic Environments. PLoS ONE.

[35] Rier ST, Kinek KC, Hay SE, Francoeur SN (2016). Polyphosphate plays a vital role in the phosphorus dynamics of stream periphyton. Freshwater Science.

[36] Crocetti GR (2000). Identification of polyphosphate-accumulating organisms and design of 16S rRNA-directed probes for their detection and quantitation. Appl Environ Microb.

[37] Munawar M, Fitzpatrick M (2007). An integrated assessment of the microbial and planktonic communities of Hamilton Harbour. Can. Tech. Rep. Fish. Aquat. Sci.

[38] Kulaev, I. S. & Vagabov, V. M. In *Advances in microbial physiology* Vol. 24 83–171 (Elsevier, 1983). 10.1016/s0065-2911(08)60385-9 6320606

[39] Faust MA, Correll DL (1977). Autoradiographic study to detect metabolically active phytoplankton and bacteria in the Rhode River estuary. Marine Biology.

[40] Currie DJ, Kalff J (1984). A comparison of the abilities of freshwater algae and bacteria to acquire and retain phosphorus1. Limnology and Oceanography.

[41] Mindl B (2005). Effects of phosphorus loading on interactions of algae and bacteria: reinvestigation of the ‘phytoplankton–bacteria paradox’in a continuous cultivation system. Aquatic Microbial Ecology.

[42] Currie DJ, Kalff J (1984). The relative importance of bacterioplankton and phytoplankton in phosphorus uptake in freshwater. Limnology and Oceanography.

[43] Saati, R. *Characterization of the Cyanobacterial Harmful Algal Bloom Community in Hamilton Harbour*, *Lake Ontario* (2016).

[44] Paerl HW, Otten TG (2013). Harmful Cyanobacterial Blooms: Causes, Consequences, and Controls. Microbial Ecology.

[45] Paerl HW, Fulton RS, Moisander PH, Dyble J (2001). Harmful freshwater algal blooms, with an emphasis on cyanobacteria. The Scientific World Journal.

[46] Molot LA, Li G, Findlay DL, Watson SB (2010). Iron‐mediated suppression of bloom‐forming cyanobacteria by oxine in a eutrophic lake. Freshwater Biology.

[47] Jiang, L. *et al*. In *Eutrophication of Shallow Lakes with Special Reference to Lake Taihu*, *China* 161–165 (Springer, 2007).

[48] Sharma K, Inglett PW, Reddy KR, Ogram AV (2005). Microscopic examination of photoautotrophic and phosphatase‐producing organisms in phosphorus‐limited Everglades periphyton mats. Limnology and Oceanography.

[49] Yuan L, Zhu W, Xiao L, Yang L (2009). Phosphorus cycling between the colonial cyanobacterium Microcystis aeruginosa and attached bacteria, Pseudomonas. Aquatic ecology.

[50] Paerl H, Lean D (1976). Visual observations of phosphorus movement between algae, bacteria, and abiotic particles in lake waters. Journal of the Fisheries Board of Canada.

[51] Currie DJ, Kalff J (1984). Can bacteria outcompete phytoplankton for phosphorus? A chemostat test. Microbial Ecology.

[52] Jansson, M. In *Phosphorus in Freshwater Ecosystems* 177–189 (Springer, 1988).

[53] Hampton SE (2017). Ecology under lake ice. Ecology Letters.

[54] Sigee, D. *Freshwater microbiology*: *biodiversity and dynamic interactions of microorganisms in the aquatic environment*. (John Wiley & Sons, 2005).

[55] Grasshoff, K., Kremling, K. & Ehrhardt, M. Methods of seawater analysis, Wiley VCH. *New York, NY* (1999).

[56] Suzumura M (2008). Persulfate chemical wet oxidation method for the determination of particulate phosphorus in comparison with a high‐temperature dry combustion method. Limnology and Oceanography: Methods.

[57] Martin P, Van Mooy BAS (2013). Fluorometric Quantification of Polyphosphate in Environmental Plankton Samples: Extraction Protocols, Matrix Effects, and Nucleic Acid Interference. Appl Environ Microb.

[58] Aschar-Sobbi R (2008). High sensitivity, quantitative measurements of polyphosphate using a new DAPI-based approach. Journal of Fluorescence.

[59] Schagerl M, Künzl G (2007). Chlorophyll a extraction from freshwater algae—a reevaluation. Biologia.

[60] American Public Health Association & American Water Works Association. In *Standard methods for examination of water and wastewater* (APHA, 1998).

[61] Thoisen C, Hansen BW, Nielsen SL (2017). A simple and fast method for extraction and quantification of cryptophyte phycoerythrin. MethodsX.

[62] Horváth H, Kovács AW, Riddick C, Présing M (2013). Extraction methods for phycocyanin determination in freshwater filamentous cyanobacteria and their application in a shallow lake. European journal of phycology.

[63] Bennett A, Bogorad L (1973). Complementary chromatic adaptation in a filamentous blue-green alga. The Journal of cell biology.

[64] Beer S, Eshel A (1985). Determining phycoerythrin and phycocyanin concentrations in aqueous crude extracts of red algae. Marine and Freshwater Research.

[65] Štrojsová A, Vrba J, Nedoma J, Šimek K (2005). Extracellular phosphatase activity of freshwater phytoplankton exposed to different *in situ* phosphorus concentrations. Marine and Freshwater Research.

[66] Herlemann DP (2011). Transitions in bacterial communities along the 2000 km salinity gradient of the Baltic Sea. The ISME journal.

[67] McMurdie PJ, Holmes S (2013). phyloseq: an R package for reproducible interactive analysis and graphics of microbiome census data. PloS one.

[68] Oksanen J (2015). Multivariate analysis of ecological communities in R: vegan tutorial. R Doc.

