# Supplementary material for: Picoplankton accumulate and recycle polyphosphate to support high primary productivity in coastal Lake Ontario
Source: Sci Rep. 2019 Dec 20;9:19563. doi: 10.1038/s41598-019-56042-5 (PMC6925121; doi:10.1038/s41598-019-56042-5)
Supplement: Supplementary file 1 — Supporting information [file 41598_2019_56042_MOESM1_ESM.pdf]

Supplementary Information for

Picoplankton accumulate and recycle polyphosphate to support high productivity in coastal Lake Ontario

Jiying Li[1*], Diane Plouchart[1], Arthur Zastepa[2], and Maria Dittrich[1]

[1]Department of Physical and Environmental Sciences, University of Toronto Scarborough, Toronto, ON, M1C 1A4, Canada

[2]Canada Center for Inland Waters, Environment and Climate Change Canada, Burlington, ON, L7S 1A1, Canada

[*]Corresponding author:

Present postal address: 2205 East 5th Street, Duluth, MN 55812, USA
Tel. +1 (218) 409-5267
Email: jiying.li@utoronto.ca

This PDF file includes:

Figs. S1 to S8
Tables S1 to S2

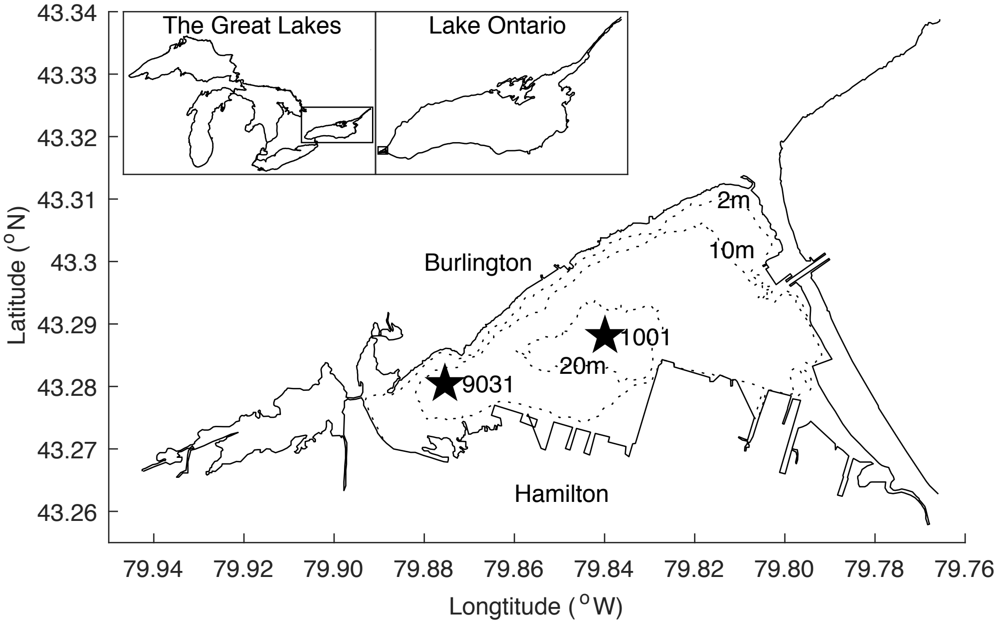

Fig. S1. Sampling locations in Hamilton Harbour, Lake Ontario. The map is modified from Dermott et al. 2007[1].

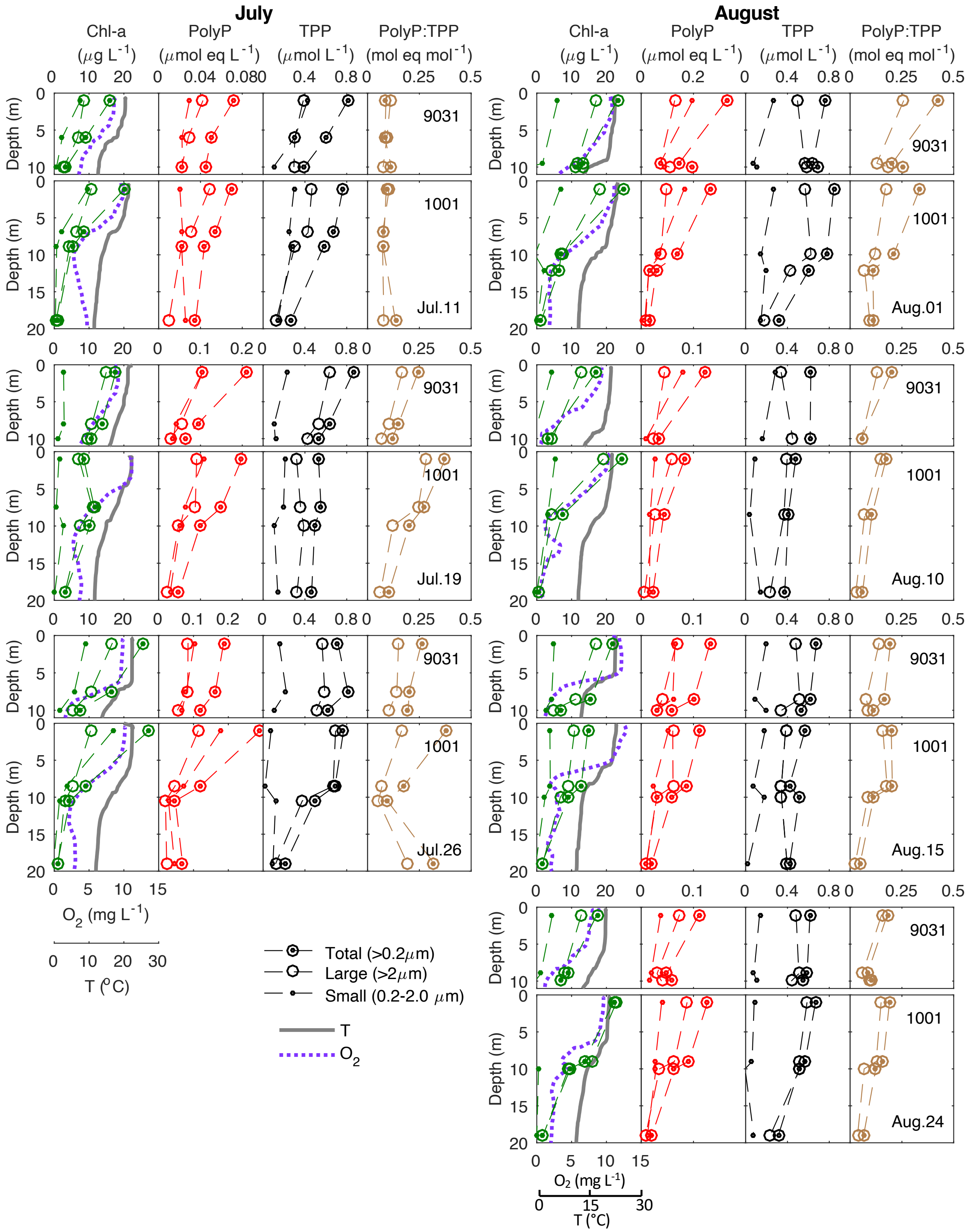

Fig. S2 Vertical distributions of temperature (T), dissolved oxygen (O_2), chlorophyll a, polyP, total particulate phosphorus (TPP), and the ratio between polyP and particulate P (polyP:PP) in the water column of Hamilton Harbour at sites 9031 (top) and 1001(bottom), in July and August, 2017. Measurements were taken at 1 m below surface, one or two locations within the thermocline, and 1 m above the bottom. Error bars (standard deviations of the mean for three replicate samples) are typically smaller than the size of the markers.

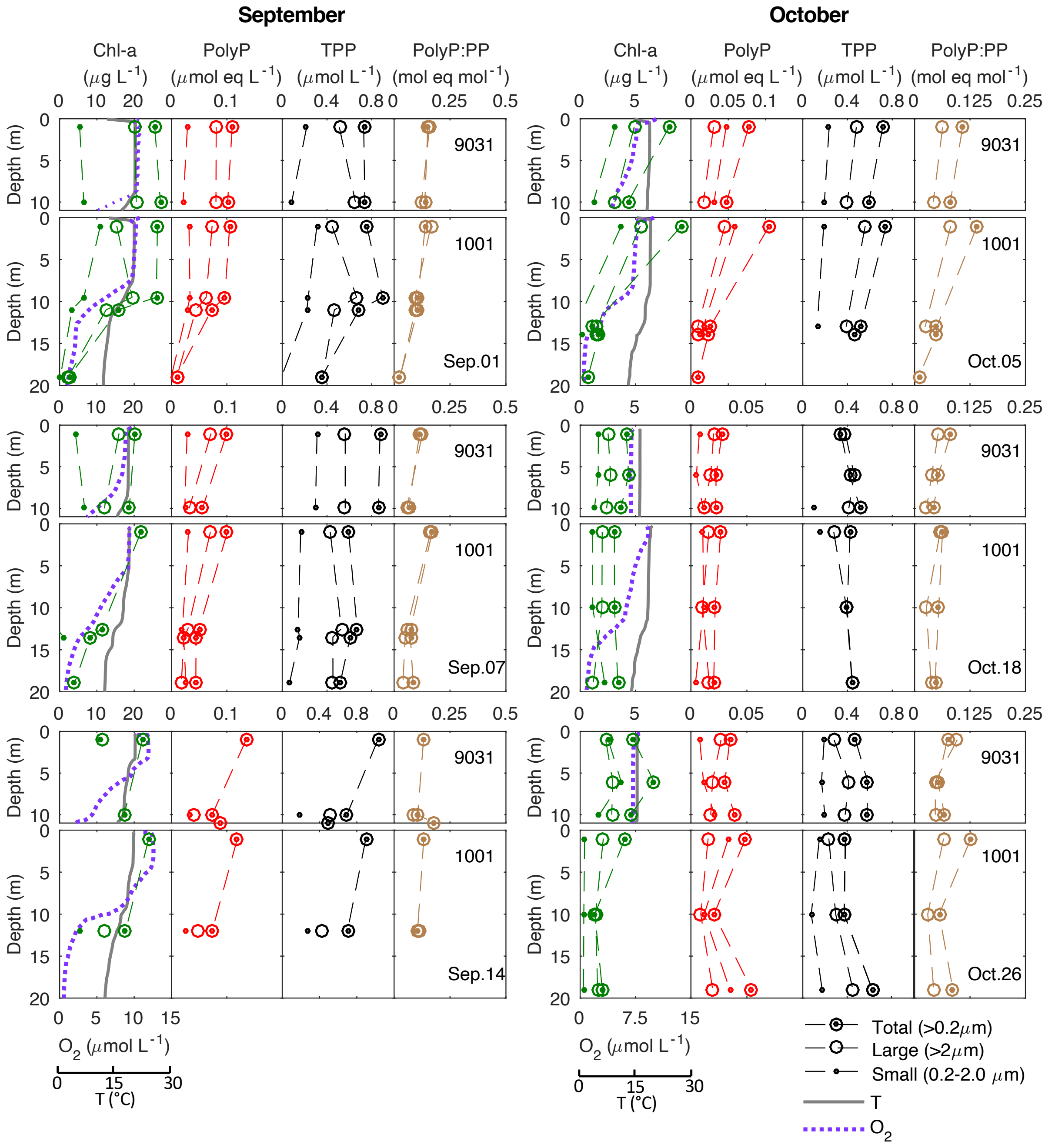

Fig. S3. Vertical distributions of temperature (T), dissolved oxygen (O_2), chlorophyll a, polyP, total particulate phosphorus (TPP), and the ratio between polyP and particulate P (polyP:PP) in the water column of Hamilton Harbour at sites 9031 (top) and 1001(bottom), in September and October, 2017. Measurements were taken at 1 m below surface, one or two locations within the thermocline, and 1 m above the bottom. Error bars (standard deviations of the mean for three replicate samples) are typically smaller than the size of the markers.

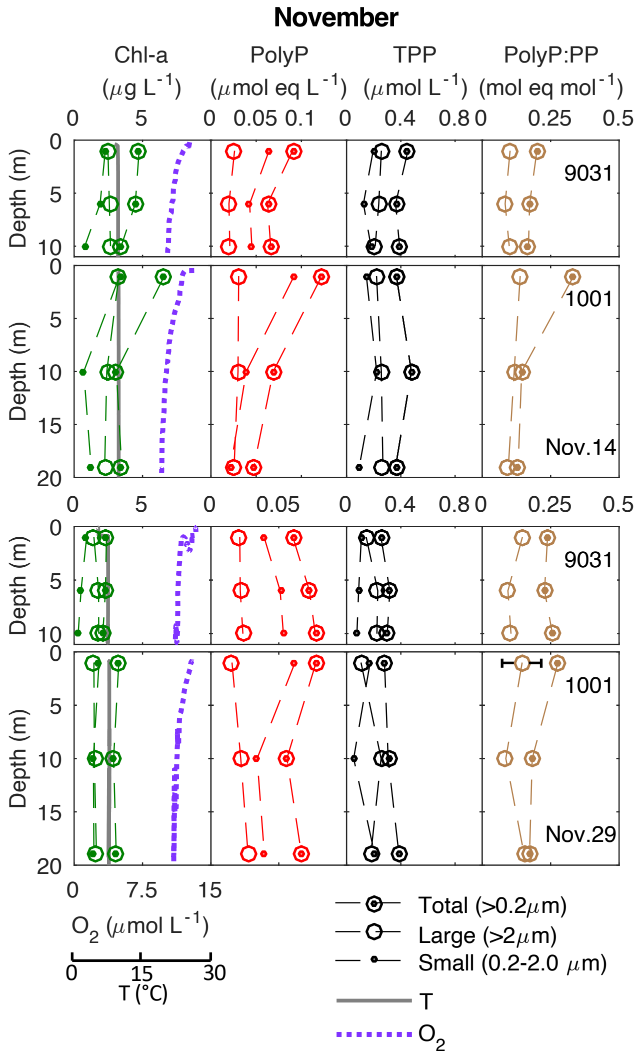

Fig. S4. Vertical distributions of temperature (T), dissolved oxygen (O_2), chlorophyll a, polyP, total particulate phosphorus (TPP), and the ratio between polyP and particulate P (polyP:PP) in the water column of Hamilton Harbour at sites 9031 (top) and 1001(bottom), in November, 2017. Measurements were taken at 1 m below surface, one or two locations within the thermocline, and 1 m above the bottom. Error bars (standard deviations of the mean for three replicate samples) are typically smaller than the size of the markers.

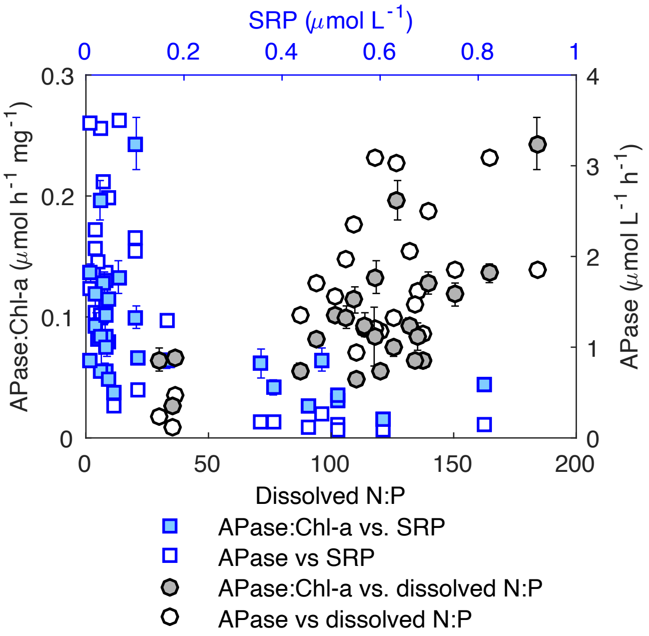

Fig. S5. Activity of alkaline phosphatase (APase and APase:Chl-a) as functions of soluble reactive phosphorus (SRP) and the ratios of dissolved N:P. Data are from both sites 9031 and 1001 for the entire sampling period, and only include measurements in the surface water. Error bars indicate standard deviations of the mean for three replicate samples and are not shown where they are smaller than marker size.

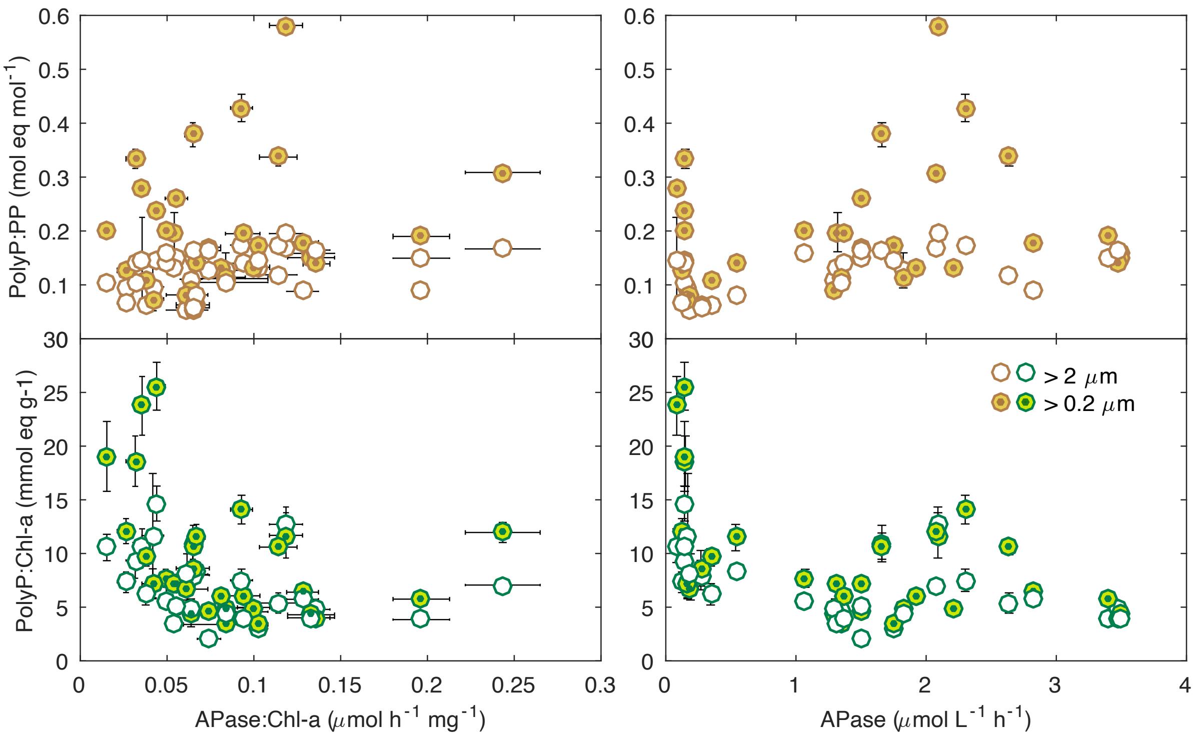

Fig. S6 PolyP ratios (polyP:PP and polyP:Chl-a) in different size fractions plotted against the activity of alkaline phosphatase (APase and APase:Chl-a). Data are from both sites 9031 and 1001 for the entire sampling period, and only include measurements in the surface water. Error bars indicate standard deviations of the mean for three replicate samples; error bars are not shown where they are smaller than marker size.

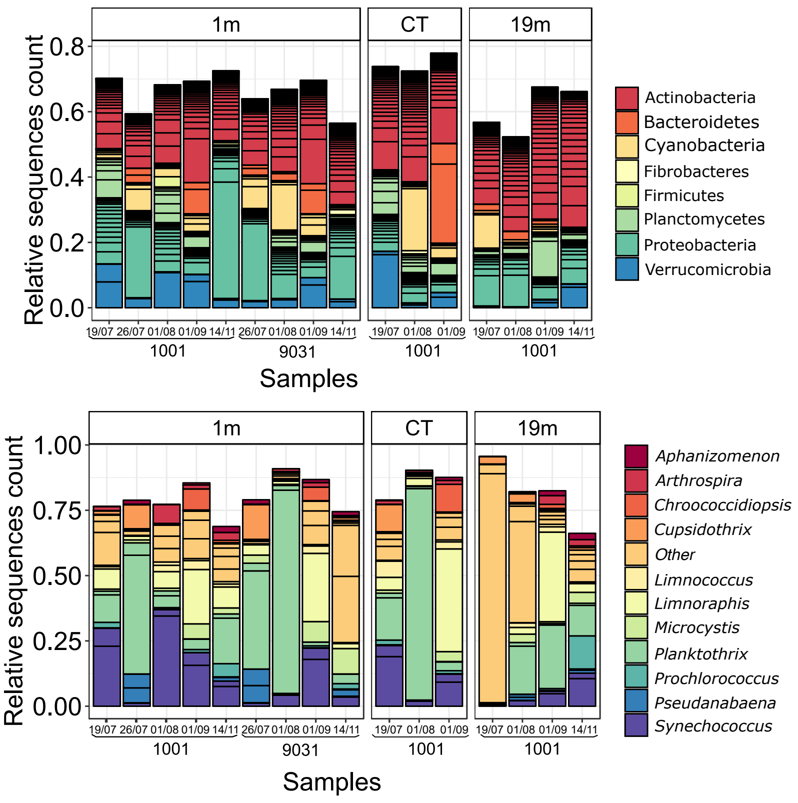

Fig. S7. The relative abundance of the eight most abundant phyla and the twelve most abundant genera of Cyanobacteria.

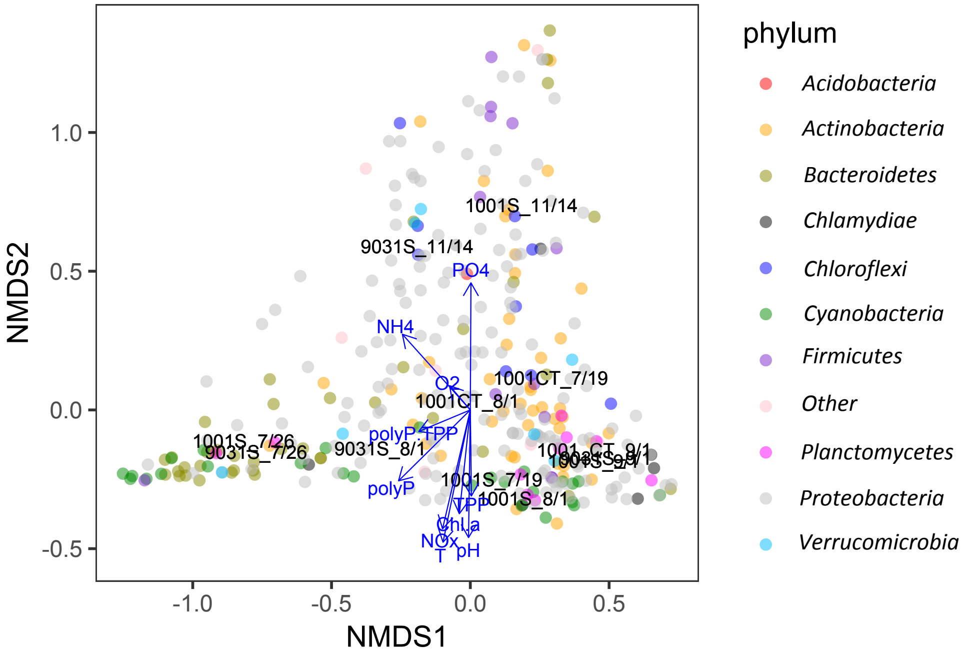

Fig. S8. Non-metric multidimensional scaling (NMDS) of the prokaryotic communities (genera) in the water column of Hamilton Harbour. Genera of different phylum groups are distinguished by colors. The arrows show the directions of the environmental factors obtained by fitting them in the ordination space of samples, and the significance of the fit are indicated by the lengths of the arrows (scaled by a factor of 0.5). The most significant variables **(**p< 0.1) are temperature, SRP (PO_4), and NH_4^+.

Table S1. Sampling information. Maximum depths are 12 m and 24 m at sites 9031 and 1001, respectively.

Date	Station	Depth (m)	Filter
07/11/2017	9031	1, 6, 10	0.2 μm GTTP; 1.2 μm RTTP
	1001	1, 7, 9, 19	0.2 μm GTTP; 1.2 μm RTTP
07/19/2017	9031	1, 8, 10	0.2 μm GTTP; 1.2 μm RTTP
	1001	1, 7.5, 10, 19	0.2 μm GTTP; 1.2 μm RTTP
07/26/2017	9031	1, 7.5, 10	0.2 μm GTTP; 1.2 μm RTTP
	1001	1, 8.5, 10.5, 19	0.2 μm GTTP; 1.2 μm RTTP
08/01/2017	9031	1, 9.5, 10	0.2 μm GTTP; 2.0 μm TTTP
	1001	1, 10, 12, 19	0.2 μm GTTP; 2.0 μm TTTP
08/10/2017	9031	1, 10	0.2 μm GTTP; 2.0 μm TTTP
	1001	1, 8.5, 19	0.2 μm GTTP; 2.0 μm TTTP
08/15/2017	9031	1, 8.5, 10	0.2 μm GTTP; 2.0 μm TTTP
	1001	1, 8.5, 10, 19	0.2 μm GTTP; 2.0 μm TTTP
08/24/2017	9031	1, 9, 10	0.2 μm GTTP; 2.0 μm TTTP
	1001	1, 9, 10, 19	0.2 μm GTTP; 2.0 μm TTTP
09/01/2017	9031	1, 10	0.2 μm GTTP; 2.0 μm TTTP
	1001	1, 9.5, 11, 19	0.2 μm GTTP; 2.0 μm TTTP
09/07/2017	9031	1, 10	0.2 μm GTTP; 2.0 μm TTTP
	1001	1, 12.5 13.5, 19	0.2 μm GTTP; 2.0 μm TTTP
09/14/2017	9031	1, 10, 11	0.2 μm GTTP; 2.0 μm TTTP
	1001	1, 12	0.2 μm GTTP; 2.0 μm TTTP
10/05/2017	9031	1, 10	0.2 μm GTTP; 2.0 µm TTTP
	1001	1, 13, 14, 19	0.2 μm GTTP; 2.0 µm TTTP
10/18/2017	9031	1, 6, 10	0.2 μm GTTP; 2.0 μm TTTP
	1001	1, 10, 19	0.2 μm GTTP; 2.0 μm TTTP
10/26/2017	9031	1, 6, 10	0.2 μm GTTP; 2.0 μm TTTP
	1001	1, 10, 19	0.2 μm GTTP; 2.0 μm TTTP
11/14/017	9031	1, 6, 10	0.2 μm GTTP; 2.0 μm TTTP
	1001	1, 10, 19	0.2 μm GTTP; 2.0 μm TTTP
11/27/2107	9031	1, 6, 10	0.2 μm GTTP; 2.0 μm TTTP
	1001	1, 10, 19	0.2 μm GTTP; 2.0 μm TTTP

Table S2. Lost of the total particulate P (TPP) and polyP in the water column, calculated by (P_S-P_B)/P_S, where P_S and P_B are the concentrations of TPP or polyP in the surface and bottom waters, respectively. Student's t-test suggests different recycling efficiency for TPP and polyP ($p < 0.001$ for both sites).

Date	Station	TPP				PolyP			
		> 0.2 μm	> 0.7 μm	>2.0 μm	Average	> 0.2 μm	>0.7 μm	> 2.0 μm	Average
07/11/2017		58%	47%	23%	43± 14%	30%	80%	48%	52± 21%
07/19/2017		38%	10%	34%	27± 13%	70%	64%	73%	69± 4%
07/26/2017		24%	28%	14%	22± 6%	35%	59%	36%	43± 11%
08/01/2017		9%	8%		8± 1%	56%	37%	43%	45± 8%
08/10/2017	9031	1%			1%	73%		45%	59±14%
08/15/2017		22%		34%	28± 6%	55%		55%	55± 0%
08/24/2017		12%		14%	13± 1%	55%		58%	57± 2%
09/07/2017						44%		55%	49± 2%
09/14/2017		32%			32%	46%			46%
10/05/2017		17%	22%	17%	19± 2%	39%	64%	44%	49±11%
**Average**			**23± 13%**				**53± 13%**		
07/11/2017		61%	76%	74%	70± 6%	30%	80%	48%	52± 21%
07/19/2017		16%		20%	18± 2%	49%	76%	81%	69± 14%
07/26/2017		72%	58%	83%	71±10%	76%	86%	78%	80± 4%
08/01/2017		66%	62%	72%	67± 4%	84%	89%	83%	85± 3%
08/10/2017		22%		41%	31± 9%	89%	93%	83%	88± 4%
08/15/2017	1001	26%			26%	73%		89%	81± 8%
08/24/2017		53%		60%	57± 3%	82%		84%	83± 9%
09/01/2017		61%		46%	53± 7%	84%		90%	88± 3%
09/07/2017		23%		14%	18± 4%	91%		87%	89± 2%
09/14/2017		37%			37%	56%		75%	65± 9%
10/05/2017						38%			38%
**Average**			**50± 22%**				**80± 13%**		

References

1 Dermott, R. *et al.* Assessment of lower food web in Hamilton Harbour, Lake Ontario, 2002–2004. *Can. Tech. Rep. Fish. Aquat. Sci* **2729**, 1-120 (2007).
